# Arsenic exposure and intestinal microbiota in children from Sirajdikhan, Bangladesh

**DOI:** 10.1371/journal.pone.0188487

**Published:** 2017-12-06

**Authors:** Xiaoxi Dong, Natalia Shulzhenko, Julien Lemaitre, Renee L. Greer, Kate Peremyslova, Quazi Quamruzzaman, Mahmudar Rahman, Omar Sharif Ibn Hasan, Sakila Afroz Joya, Mostofa Golam, David C. Christiani, Andriy Morgun, Molly L. Kile

**Affiliations:** 1 College of Pharmacy, Oregon State University, Corvallis, OR, United States of America; 2 College of Veterinary Medicine, Oregon State University, Corvallis, OR, United States of America; 3 Dhaka Community Hospital, Wireless Railgate, Dhaka, Bangladesh; 4 Department of Environmental Heath, Harvard TH Chan School of Public Health, Boston, MA, United States of America; 5 School of Biological and Population Health Sciences, College of Public Health and Human Sciences, Oregon State University, Corvallis, OR, United States of America; University of Rajshahi, BANGLADESH

## Abstract

**Background:**

Arsenic has antimicrobial properties at high doses yet few studies have examined its effect on gut microbiota. This warrants investigation since arsenic exposure increases the risk of many diseases in which gut microbiota have been shown to play a role. We examined the association between arsenic exposure from drinking water and the composition of intestinal microbiota in children exposed to low and high arsenic levels during prenatal development and early life.

**Results:**

16S rRNA gene sequencing revealed that children with high arsenic exposure had a higher abundance of *Proteobacteria* in their stool compared to matched controls with low arsenic exposure. Furthermore, whole metagenome shotgun sequencing identified 332 bacterial SEED functions that were enriched in the high exposure group. A separate model showed that these genes, which included genes involved in virulence and multidrug resistance, were positively correlated with arsenic concentration within the group of children in the high arsenic group. We performed reference free genome assembly, and identified strains of *E*.*coli* as contributors to the arsenic enriched SEED functions. Further genome annotation of the *E*.*coli* genome revealed two strains containing two different arsenic resistance operons that are not present in the gut microbiome of a recently described European human cohort (Metagenomics of the Human Intestinal Tract, MetaHIT). We then performed quantification by qPCR of two arsenic resistant genes (ArsB, ArsC). We observed that the expression of these two operons was higher among the children with high arsenic exposure compared to matched controls.

**Conclusions:**

This preliminary study indicates that arsenic exposure early in life was associated with altered gut microbiota in Bangladeshi children. The enrichment of *E*.*coli* arsenic resistance genes in the high exposure group provides an insight into the possible mechanisms of how this toxic compound could affect gut microbiota.

## Introduction

Globally, it is estimated that 100 million people are exposed to elevated levels of arsenic from drinking contaminated water [1]. One country that is heavily affected by arsenic-contaminated drinking water is Bangladesh [2]. Chronic arsenic exposure is known to cause skin lesions and cancer [3] and this data have been used in risk assessment to establish maximum allowable contaminant levels in drinking water [4]. There is also considerable evidence that chronic arsenic exposure increases the risk of type 2 diabetes [5] and cardiovascular disease [6]. Furthermore, recent studies have shown that arsenic exposure during development and early childhood increases the risk for respiratory and diarrheal infections in children [7, 8].

Arsenic influences multiple disease pathways [9]. While no scientific consensus has been achieved regarding arsenic’s mode of action, there is evidence that it exerts its toxicity through oxidative stress [10], epigenetic modification [11], and altering signal transduction pathways [12]. Recently, arsenic’s ability to affect microbes has been proposed as a possible mode of action [13] yet there have been very few studies examining the effect of arsenic on the microbiome of humans [14]. This is somewhat surprising given that arsenic is known to have antimicrobial properties and was used to treat infectious diseases in the pre-antibiotic era [15]. Furthermore, the role of microbes -and specifically gut microbiota- on human health has exploded in the past decade and there are reports demonstrating the mechanistic role of gut microbiota in cancer[16], type 2 diabetes [17], cardiovascular disease [18], and infectious diseases [19]—the same diseases that are increased in populations exposed to chronic arsenic exposure.

Given the primary route of arsenic exposure in humans is ingestion of contaminated drinking water and/or food [20, 21], the gut microbiota may be the most susceptible to arsenic exposure; thus playing a role in arsenic-related diseases. Furthermore, arsenic in drinking water crosses the placenta [22] but arsenic is not transmitted in breast milk [23]. This leads to discrete exposure periods during gestation and early childhood. Since the gut is colonized immediately after birth [24], it is important to understand the potential for arsenic exposure to affect children’s gut microbiota using measures of arsenic exposure near the time of delivery. Therefore, the overall goal of this study was to conduct a preliminary investigation that evaluated the association between arsenic exposure and intestinal microbiota in children who resided in an arsenic-endemic region of Bangladesh. We hypothesize that the composition of intestinal microbiota would be different in children who had high arsenic exposure during the perinatal and prenatal period compared to children matched by age, sex, and residence that had low arsenic exposure at this same developmental time point.

## Results

The mean drinking water arsenic concentration for the high and low exposed groups was 218.8 μg/L (Standard deviation, SD: 166.1 μg/L) and 1.7 μg/L (SD: 1.9 μg/L), respectively. Aside from arsenic exposure, there was no significant difference between the high and low exposed groups based on gender, age, body mass index, or mid-arm circumference (Table 1).

**Table 1:** Selected characteristics of 50 children included in this nested case-control study.

	High Arsenic (N = 25)	Low Arsenic (N = 25)	
	Mean (SD)	Mean (SD)	p-value
Age (yrs)	4.6 (1.7)	4.4 (1.2)	0.63^a^
Body Mass Index (kg/m^2^)	14.3 (1.7)	14.9 (1.5)	0.25 ^a^
Mid Arm Circumference (cm)	13.0 (1.1)	13.4 (1.4)	0.14 ^a^
Water arsenic (μg/L)	218.8 (166.1)	1.7 (1.9)	<0.0001^b^
	Frequency	Frequency	
Gender (females)	48%	48%	1.0^c^

^a^ p-value obtained using a two-tailed t-test

^b^ p-value obtained using the Wilcoxon signed-rank test

^c^ p-value obtained from a χ^2^ test

### Microbiota composition

To determine if arsenic exposure status measured during the prenatal and perinatal period influenced gut microbial composition, we performed sequencing of bacterial 16S rRNA gene in fecal DNA. Overall, the gut microbial compositions in the high and low exposure group were similar with most prevalent phyla being Bacteriodetes and Firmicutes, which has been reported in other populations [25]. Analysis of alpha and beta diversity of microbiota did not indicate global differences in bacterial communities between high and low arsenic exposure groups (S1 Fig).

**Fig 1:**
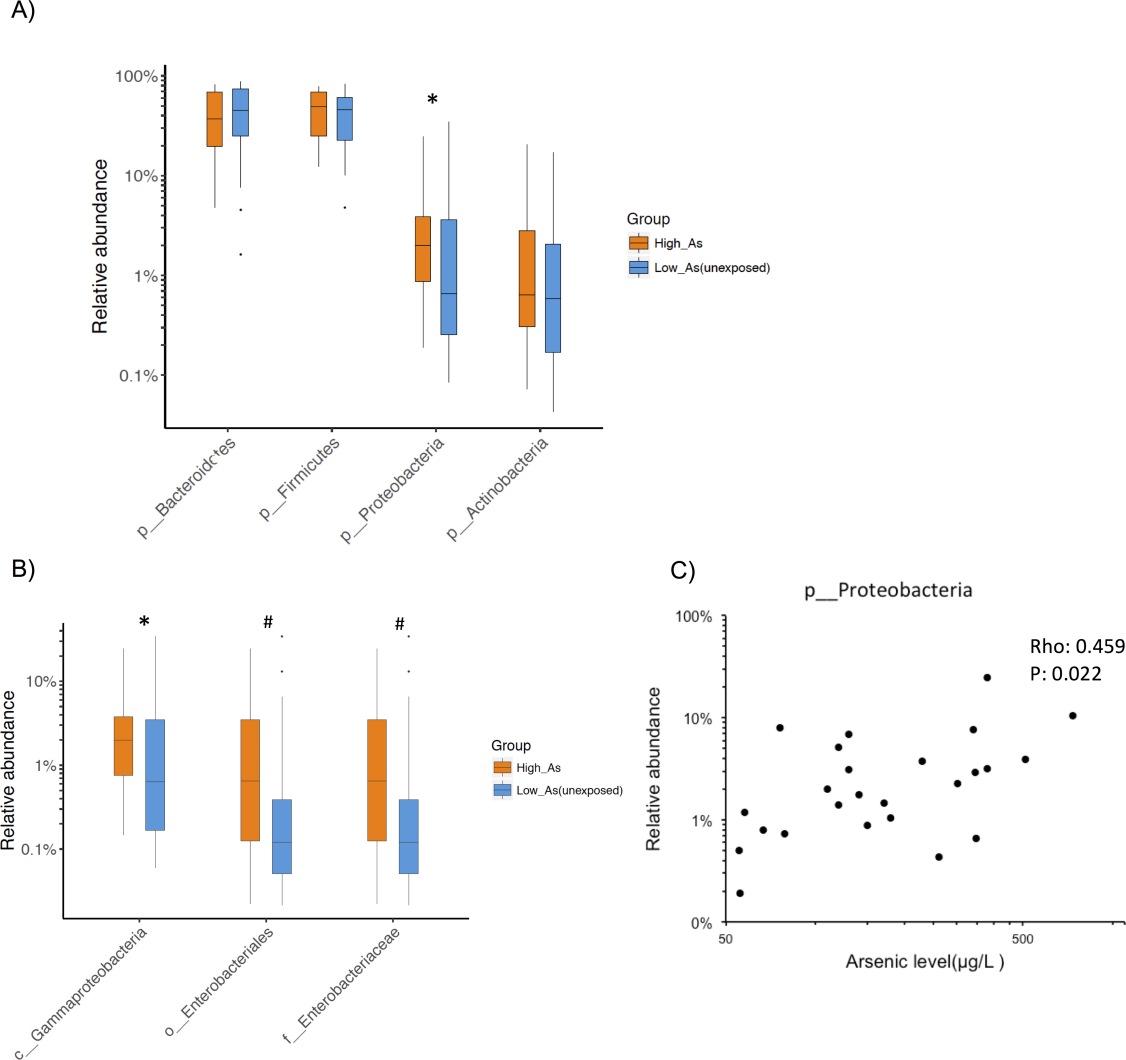
Microbial taxonomic composition in high arsenic exposure group and low arsenic exposure group based on 16S rRNA V4 gene region sequencing. (A) Relative abundance of phyla for high arsenic exposure samples (n = 25, orange color) and low exposure samples (n = 25, blue color). The boxplot represent interquartile range with the black bar indicating the median relative abundance and error bars represent minimum and maximum values. Outliers are represented by solid circles. *P<0.02, Mann–Whitney U test. (B) Relative abundance of three lower taxonomic ranks of phylum *Proteobacteria* for high arsenic exposure and unexposed samples. * P<0.03, # P<0.1(C) Correlation between relative abundance of phylum *Proteobacteria* and water arsenic level in high arsenic exposure group. p-value is calculated for two-tailedSpearman correlation coefficient.

Next, we compared microbial frequencies between the high and low arsenic exposure groups. We observed an increased abundance of phylum *Proteobacteria* in the high arsenic exposure group compared to the matched low arsenic exposure group (two-tailed P<0.02, false discovery rate (FDR) 0.1, considering phyla with >1% of abundance, Fig 1A). Deeper inspection at other taxonomic levels revealed additional trends where the high arsenic group had increased relative abundance of the class *Gammaproteobacteria* (two-tailed P<0.03, FDR 0.6), order *Enterobacteriales* (p<0.1, FDR 0.7), family *Enterobacteriaceae* (two-tailed P<0.1, FDR 0.7) compared to the matched low exposure group (Fig 1B). Notably, all these three taxonomic groups belong to the phylum *Proteobacteria*.

A sub-analysis conducted within the high arsenic exposure group demonstrated a positive correlation between the abundance of *Proteobacteria* and the concentration of arsenic measured in drinking water expressed as a continuous variable (Fig 1C). Additionally, three other taxa within this phylum showed a weaker positive correlation with arsenic levels (data not shown). Thus, two separate statistical analyses–where microbial differences were evaluated between high and low arsenic exposure groups and where microbial abundance was correlated with arsenic concentrations expressed as a continuous variable–showed that arsenic exposure was related to the composition of gut microbiota. We only conducted the correlation analysis among the high arsenic exposure group because this group contained a range of arsenic values. The vast majority of the arsenic concentrations in the low arsenic exposure group were below the limit of detection. It was not possible to combining both groups due to the strong underlying biomodal distribution in arsenic exposure which was a feature of the recruitment strategy and resulted in heteroscedasticity.

### Metagenomic composition

**Fig 2:**
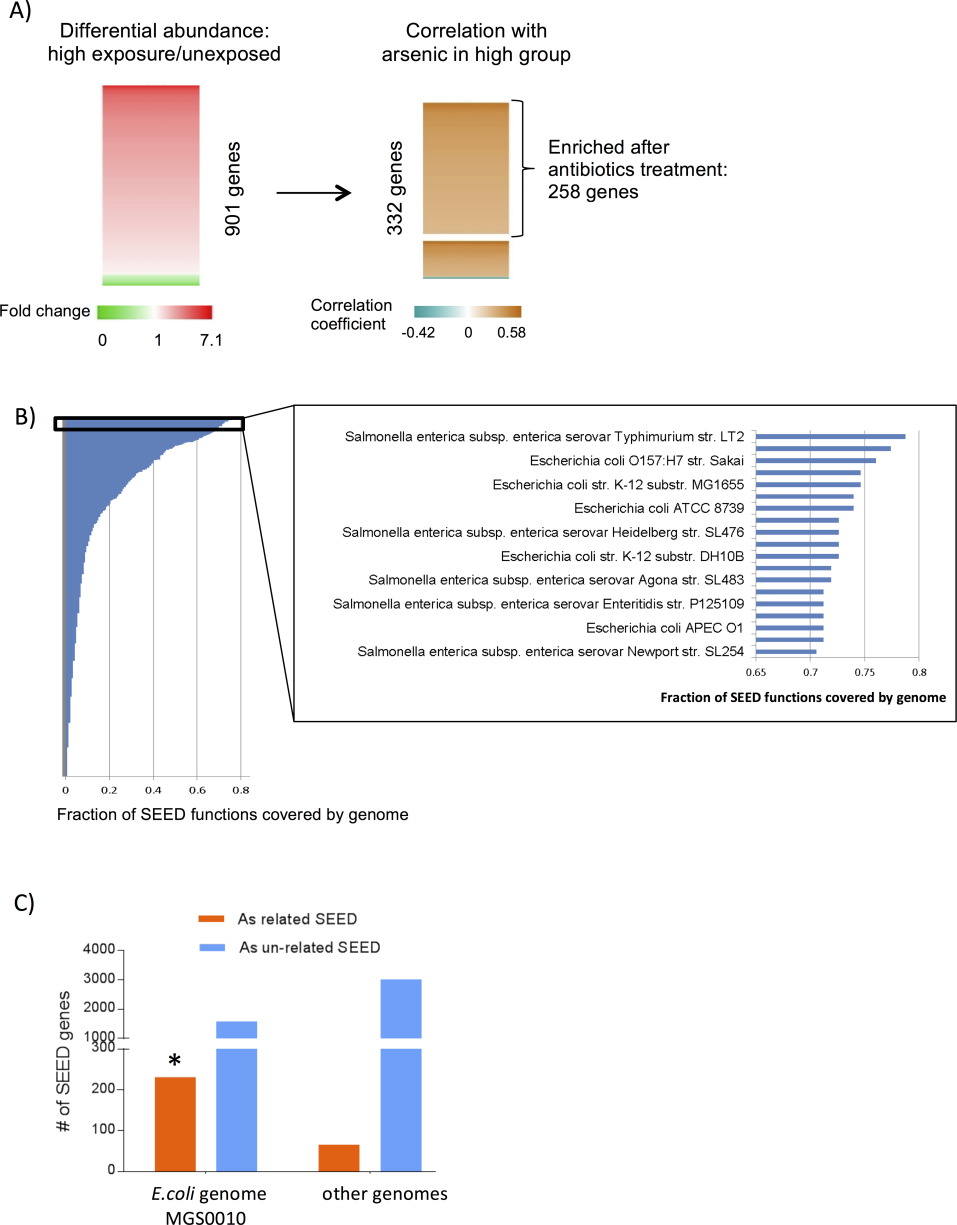
Whole genome shotgun sequencing detected microbial SEED functions associated with arsenic level. (A) Heatmap showing fold change of 901 different abundant SEED functions between high exposure versus low exposure group and correlation coefficients (Spearman) with arsenic levels in high exposure group for 332 SEED functions. The 332 SEED functions either have higher abundance in high exposure group and positively correlated with arsenic level in high exposure group or have lower abundance in high exposure group and negatively correlated with arsenic level in high exposure group (Fisher combined FDR <0.1). Brackets mark 258 SEED functions that also had increased relative abundance in mice after treatment by four antibiotics (ampicillin, neomycin trisulfate, vancomycin, metronidasole) [27]. (B). Fraction of the 332 overlapping SEED functions covered by reference microbial genomes. Those covering more than 70% of 332 gene set are listed. C) Distribution of Arsenic related and non-related SEED genes in assembled genome MGS0010 (*E*.*coli*) and other genomes. *Chi-squared test odds ratio 6.69, FDR 1.08e-48.

For analysis of genomic content, we first inferred Kyoto Encyclopedia of Genes and Genomes (KEGG) function information from 16s rRNA sequencing data using PICRUST (Phylogenetic Investigation of Communities by Reconstruction of Unobserved States)[26]. Comparison between high and low arsenic exposure groups revealed no differentially abundant KEGG functions (S1 Table). Since shotgun whole genome sequencing provides actual genome content information, we then inferred SEED functions[27] from this data by mapping reads to nr protein database and using MEGAN4 (MEtaGenome Analyzer) [28] to map to SEED functions. We then compared SEED functions between high and low arsenic exposure groups to evaluate the association between arsenic exposure and taxonomic composition. This analysis identified 901 bacterial genes with differential abundance (two-tailed P <0.1) (Fig 2A, left; S2 Table) that was dependent upon arsenic exposure status. We then examined the correlation between the 901 bacterial genes and arsenic concentrations in drinking water among the high arsenic exposure group. We are interested in bacterial genes that were either 1). enriched in the high arsenic exposure group and positively correlated with arsenic concentration in drinking water, or 2). had lower abundance in high exposure group and negatively correlated with arsenic level in high exposure group. By combining statistical analysis result from class comparison and correlation, we identified a set of 332 microbial genes to be associated with arsenic exposure (FDR <0.1; S2 Table; Fig 2A, right), and we refer to this as the arsenic-enriched gene set in the following analyses.

Focusing on this arsenic-enriched subset of 332 microbial genes, we then investigated which genomes were contributing the arsenic enriched SEED functions. For this analysis, we matched the 332 bacterial genes to fully sequenced bacterial genomes available at NCBI and ranked the genomes by the proportion of 332 genes they contained (Fig 2B, left). Top ranking genomes were *Escherichia coli* and *Salmonella enterica* with over 70% of the 332 seed genes corresponding to the genomes of these two species (Fig 2B, right). To avoid bias in the reference genome database used in this analysis, we assembled genomes of MGS (metagenomic species) from our samples using a reference free approach (S3 Fig) that bins genes from the same genome together based on their similar abundances across multiple samples[29]. For the assembled 100 large genomes (number of ORFs (Open Reading Frames) in the genome >97, S7 Table), we annotated the ORFs in the genome with SEED functions. Then, we examined if the functions in each genome showed enrichment with the subset of 332 arsenic related SEED functions. From this analysis, one genome MGS0010 showed a significant tendency for enrichment within the 332 arsenic related SEED functions compared to all other genomes (odds ratio 6.69, FDR 1.08e-48) (Fig 2C, S8 Table). This enrichment remained significant when the different level of genome completeness was controlled (data not shown). Taxonomy assignment of the genome revealed that the genome was assigned to *E*.*coli* (>90% of the ORFs in the genomes were assigned to species *E*.*coli*).These results suggest that *E*. *coli* is the genome that contributed to the SEED functions enriched among the high arsenic exposure group.

**Fig 3:**
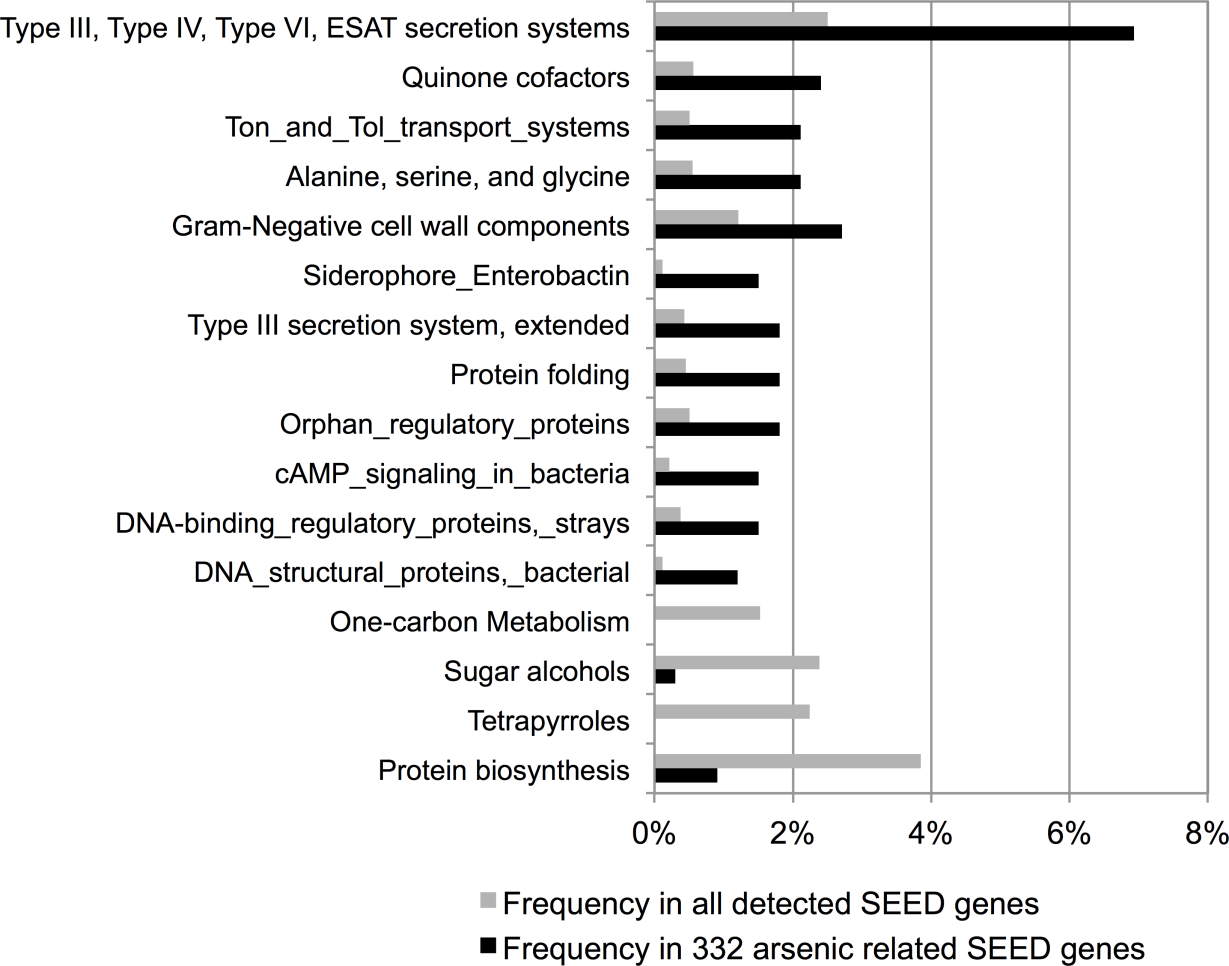
Annotations of SEED functions associated with arsenic exposure. Frequency of SEED genes annotated under specific SEED level 2 function categories within 332 arsenic related SEED functions (black color) and all SEED functions detected in the samples. The SEED level function categories in the figure are significantly overrepresented or depleted in the 332 arsenic related functions (chi-square test P value <0.05).

These data are consistent with our previous taxonomy analysis (Fig 1) and indicate that *E*.*coli* in the *Enterobacteriacea* family was associated with children exposed to high arsenic exposure during the perinatal and prenatal period compared to matched controls who were exposed to low arsenic exposure during this same developmental window. The higher SEED level categories over-represented in 332 gene set included transporter and secretion systems, quinone (oxidation-reduction) factors and siderophores among others (Fig 3). Notably, several of these proteins are known to be involved in virulence and multidrug resistance (S2 Table), including antibiotic-resistance reported for TolC protein [30].

### Antibiotic resistance

It has also been reported that microbes exposed to arsenic can acquire resistance for antibiotics [31]. To examine this possibility, we compared the genes in the arsenic enriched subset (322 genes) with genes detected in an experimental mouse system that was enriched by antibiotics (1618 genes) [32]. We observed that 78% of arsenic-enriched genes (258 genes) were also found in the bacteria surviving long-term antibiotic treatment in the mouse gut (Fig 2A; S3 Table). This association was still significant when controlling for the *E*.*coli* genome as a confounding factor (S4 Table). Using CARD (Comprehensive Antibiotic Resistance Database) database[33], we found three known antibiotic resistance genes (ermB, qnrS8, cfxA4) had a moderately higher abundance in the arsenic-enriched subset but no statistically significant associations were observed (S6A Fig). We also examined the correlation between arsenic exposure expressed as a continuous variable with the 258 genes. This analysis showed three additional antibiotic-resistance genes (soxS, marA, cpxR) tended to be positively correlate with arsenic levels. By integrating the group comparison and the correlation results, we have tentative results that suggest that the six genes are possibly related to arsenic exposure (combined Fisher P of <0.05, FDR 0.89). These results should be used to generate hypotheses for further study.

For a targeted search of arsenic metabolism related microbial genes, we mapped whole genome shotgun sequencing reads to known genes involved in arsenic metabolism genes (Arsenic efflux pump protein (ArsB), arsenic resistance protein ArsH, arsenical pump-driving ATPase (EC 3.6.3.16) (arsA), arsenical resistance operon repressor (arsR), arsenical resistance operon trans-acting repressor ArsD, arsenical-resistance protein ACR3) and compared their abundance between high and low arsenic groups. Although the difference did not reach statistical significance, there was a moderate increase in abundance of ArsB (arsenic efflux pump protein), ArsC (arsenate reductase), and ACR3 (arsenical resistance protein) in the high exposure group (data not shown).

**Fig 4:**
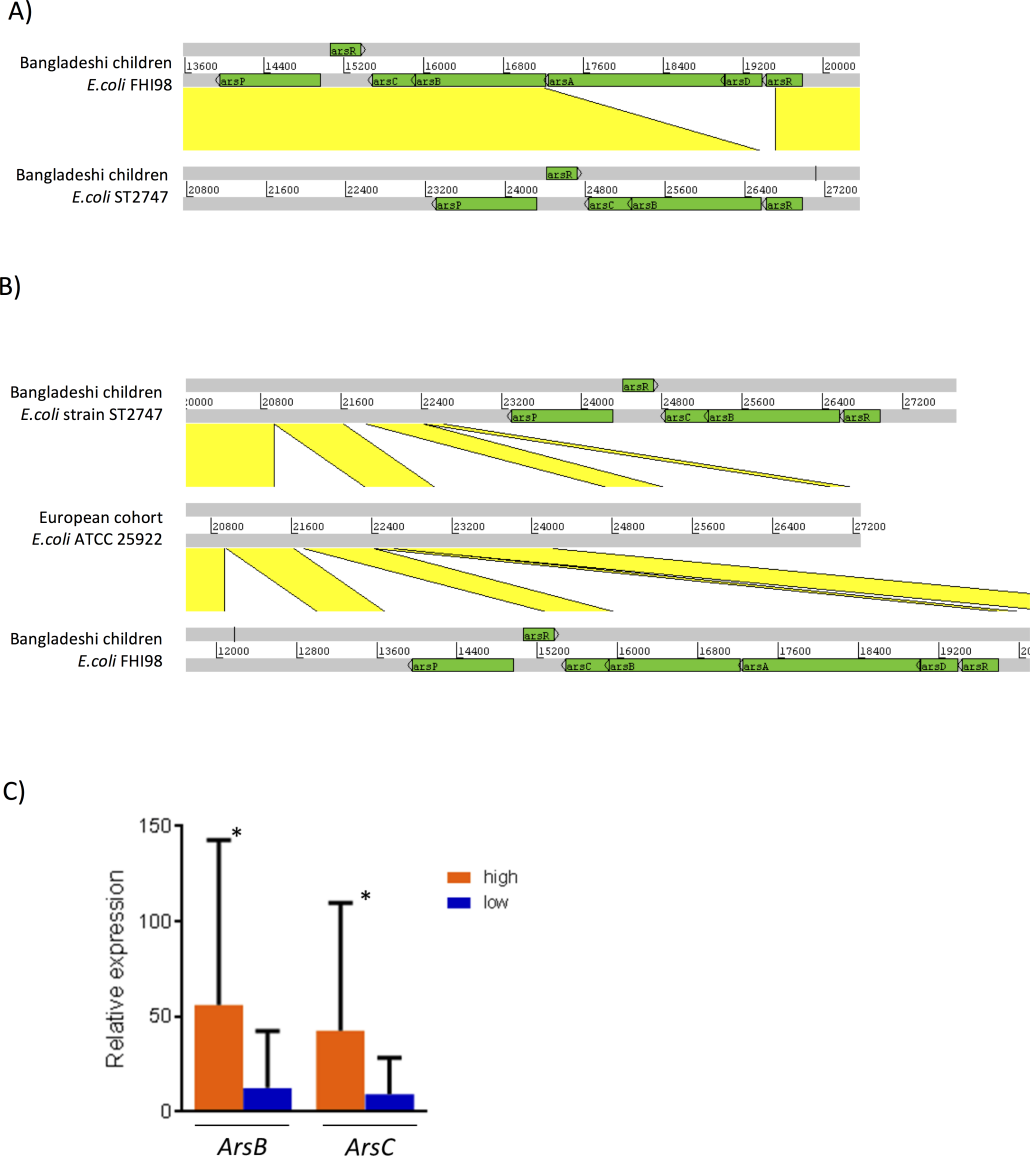
A) Comparison (displayed using Artemis Comparison Tool; doi 10.1093/bioinformatics/bti553) of DNA sequences of *E*.*coli* strains in Bangladeshi children cohort. Gray bars represent the forward and reverse strands of DNA. The yellow lines between the sequences represent existence nucleotide similarity (blastn). Arsenic resistant operons are given green color. B) Comparison of DNA sequences of *E*.*coli* strains in Bangladeshi children and European cohort. Comparison is displayed for two *E*.*coli* strains in Bangladesh children (ST2747 and FHI98) and their most similar *E*.*coli* strain in European cohort (ATCC 25922). C) qPCR quantification of two arsenic resistance operon genes (ArsB, ArsC) found in Bangladeshi children *E*.*coli* strain ST2747. (* p value<0.05).

To better characterize arsenic resistant/metabolism related genetic determinants in the gut microbiome, we annotated the assembled genomes with arsenic metabolism related genes in BacMet (Antibacterial Biocide and Metal Resistance Genes Database) database[34] and identified multiple arsenic resistance operons (S6 Table). Two of the operons ArsRDABCRP and ArsRBCRP (Fig 4A) were from the *E*.*coli* genome (MGS0010) that was in the arsenic-enriched subset of 332 arsenic related SEED functions. Through blastn search against NCBI nucleotide database, the two contigs (44.fastq_1_63:C75111 and 27.fastq_1_31:C431936) that harbor those two different arsenic resistance operons, were assigned to *E*.*coli* strains FHI98 (gi|675817476|emb|LM997367.1) and ST2747 (gi|595597955|gb|CP007392.1), respectively (99% sequence identity covering 100% contig sequences) (Fig 4A, S5 Table).

Finally, we conducted an ecological analysis where we compared the two *E*.*coli* strain contigs found in the Bangladeshi children (from both high exposure and low exposure groups)with the contigs in the gut microbiome from a European cohort [34] that does not experience the same level of arsenic exposure as Bangladesh. We found that many genes (ArsR, ArsB, ArsC, ArsP, ArsA, ArsD) in the arsenic resistance operons of *E*.*coli* in Bangladeshi children cohort were not found in their closest *E*.*coli* contig in the European cohort (no homologs coding region with >95% sequence identity, Fig 4B). This suggests arsenic adapted *E*.*coli* strains colonization in the Bangladeshi children cohort.

We then designed primers for ArsB and ArsC genes in one of the *E*.*coli* Ars operons (ArsRBCRP from strain ST2747) to quantify their levels. The result shows that the abundances of both ArsB and ArsC were significantly higher in the high arsenic exposed group than that in the low arsenic exposure group (Fig 4C).

## Discussion

Our study provides novel evidence that arsenic exposure is associated with changes in the gut microbiota in Bangladeshi children. Specifically, we observed that species within the *Proteobacteria* phylum, *Enterobacteriaceae* family, predominantly arsenic resistant *E*. *coli*, were more abundant in children who had high arsenic exposure during the prenatal and perinatal period.

We acknowledge that this is a preliminary study, and the results need to be confirmed with a larger sample size and repeated measurements of exposure. Nevertheless, even this preliminary investigation allowed us to pinpoint some alterations in microbiota with potential mechanistic implications such as the identification of arsenic resistance operons in two *E*. *coli* strains (Fig 4). Importantly, genes related to arsenic detoxification (ArsC, ArsB) were more abundant in the gut of children that had high exposure during gestation and the perinatal period–a time when the gut microbiome is first colonized–compared to children that had low arsenic exposure and were matched by age, sex, and location (Fig 4C). In addition, these results were consistently observed when we conducted two independent analyses (group comparison, and correlation analysis among the high exposure group. Also, we observed reasonably good agreement of taxa profiling between the two different molecular techniques, 16S rRNA gene and shotgun sequencing (data not shown).

While we are not aware of prior gut microbiome analysis in children who have been exposed to arsenic during the perinatal period, there is data from animal models and ecological studies that demonstrate that arsenic alters microbiota. For example, Lu *et al* observed that mice given oral exposure to 10 ppm arsenite for 4 weeks resulted in changes in composition of gut microbiota [13]. However, the changes in the microbial communities observed by Lu *et al* were different than those observed in our study. This could be attributed to at least two possible factors. First, while we studied chronic arsenic exposure that occurred during early development, Lu *et al* studies acute high arsenic exposure in adult animals. These different exposure scenarios would place gut microbiota under two very different toxic gradients. Secondly, the dissimilarities between our and Lu *et al*’s results could be attributed to the differences between human and mouse gut microbiota. Interestingly, an ecological study that examined soil microbial communities found an increased proportion of *Proteobacteria* in arsenic contaminated soil which corresponds our observation of increased *Proteobacteria* in gut microbiota of arsenic-exposed children [35]. Notably, besides observing a similarity to our results at taxonomic level, this study reported an increased nucleic acid sequence diversity of ACR3, a gene involved in arsenic metabolism.

A surprising observation of our study is a striking resemblance between the gene content of microbiota associated with arsenic exposure and antibiotic-resistant microbiota that we previously observed in a mouse model [32]. Although the connection between environmental arsenic contamination and antibiotic resistance has been previously reported [36], our results provide a novel view on this problem. While our analyses demonstrated few antibiotic-resistance genes (6 out of 3006 genes from antibiotics-resistance CARD database [33]) increased in arsenic-associated microbes, the overall similarity of gene content between antibiotic and arsenic enriched microbiota is very high (Fig 2A). Such a strong resemblance can potentially be explained by the fact that arsenic may select microbes containing genes with general resistance to different toxic compounds (such as efflux pump TolC) including metals and antibiotics.

Among limitations of our study which may influence our interpretation of these results are unmeasured confounders. For instance, arsenic exposure is associated with increased infections in children [7, 8] and thus might be leading to higher incidence of antibiotic use. While we did ask parents about their children’s medical histories and noted that no participants were currently taking any medication, we cannot completely rule out antibiotics as a confounder as medication histories might be subject to recall bias. Also, we assigned children into the two exposure categories based on the amount of arsenic measured in their household’s drinking water. We did not have biomarkers of exposure to confirm exposure status. However, prior studies in rural Bangladesh populations have demonstrated that arsenic concentrations in drinking water are highly correlated with biomarkers of internal dose including toenails [37] and hair [38].

Additionally, we did not control for dietary factors which could influence gut microbiota. However, for this to be an alternative explanation for our findings, the dietary factor would have to be highly correlated with the concentration of arsenic in maternal drinking water during pregnancy. It is also unknown if the observed shift in gut microbiome is a feature of exogenous pressures on soil bacteria which then colonizes the gut since it has been shown that *Proteobacteria* are more abundant in arsenic-contaminated soil [35]. However, it is plausible that bacterial communities have a similar response to arsenic whether their niche is the gut or soil.

While confirmation of our results is needed in independent cohorts, there are several important questions that still need to be addressed. For instance, we categorized this population based on prenatal arsenic exposure levels and examined the microbiota in children up to 6 years later. Because the exposure was examined prospectively, it is not possible to ascertain whether the observed microbiome alternations were due to colonization at birth, during early childhood, or a function of continuous arsenic exposure. It is likely that children would have been exposed through drinking water and dietary sources upon weaning [39]. Given the dynamic changes that occur in gut microbiota [24], it would be interesting to collect fecal samples from the mother during the prenatal period and repeated samples from both the mother and child during early childhood to answer this question. Another interesting approach would be to examine the role of breastfeeding since it is known that arsenic is not transmitted through breast milk [23]. Despite the effects of breastfeeding on gut microbiome [40], comparing breastfed to bottle fed infants could potentially isolate the effect of arsenic exposure on gut microbiome.

Furthermore, a recent study on microbiome of children from developing countries all over the world including Bangladeshi found that children with moderate/severe diarrhea had higher proportion of members of *Escherichia* and *Shigella* compared to controls [41]. In addition, members of *Enterobacteriaceae* were previously detected in higher proportions in the gut microbiota of malnourished Bangladeshi children but the relation to arsenic exposure was not investigated [42, 43]. Importantly, none of the children in our study had evidence of malnutrition and had body mass index z-scores that were +/- 2 standard deviations of age and gender adjusted norms, ruling it out as a confounding factor.

Finally, we were able to detect *E*.*coli* strains that harbor arsenic resistance operons and those were not detected in the gut microbiome of European cohort [34] that do not live in highly arsenic contaminated regions. This is in agreement with the general idea that gut microbiome is influenced by the environment the host is exposed to. In addition, the observation of higher abundance of arsenic detoxification genes (ArsB, ArsC) in high exposure groups indicate the cells possess arsenic resistant potential could have higher fitness when exposed to this xenobiotics. Moreover, the arsenic resistant system (ArsRBC), when working, by reducing As(V) into As(III) via a cytoplasmic arsenate reductase (ArsC) and extruding the latter from the cellular compartment by means of a membranous As(III) efflux pump (ArsB). This possibly would affect both other members of the microbiome and host through more available arsenic they are in contact with. This sheds light on how xenobiotics could shape microbiome composition and how hosts with different microbiomes could respond differently to xenobiotics.

## Conclusion

Our results indicate that environmentally-relevant levels of arsenic were related to altered gut microbiota in children which is consistent with recent evidence in mouse models. Given the importance of gut microbiota to human health, further research is needed to confirm these preliminary results and determine the contribution of gut microbiome to arsenic-related diseases.

## Methods

### Study population

In 2013, we conducted a nested study (N = 50) that recruited participants from a prospective birth cohort that was established in Bangladeshi (2007–2011). Specifically, we recruited 25 children aged 4–6 years old who were exposed to high arsenic exposure during development and in early life and matched them on sex, location of residence, and age (within 6 months) with children who had low arsenic exposure during development and early life. All participants were recruited from within a 15 kilometer radius of Sirajdikhan, Bangladesh (23.5962° N, 90.3937° E) and were selected based on arsenic exposure measured in the household’s drinking water when the mother was enrolled in the birth cohort (< 28 weeks gestational age). Overall, the level of arsenic in this region spans a wide range. Arsenic concentrations were measured in the drinking water of the 307 participants in the larger cohort recruited in Sirajdikhan. The median arsenic concentration in this group was 1.3 μg/L but approximately 26% of the households exceeded the Bangladesh drinking water standard of 50 μg/L.

In this nested study, we defined high arsenic exposure as the household’s drinking water well contained > 50 μg/L and low arsenic exposure was defined as the household’s well contained <10 μg/L. Each household has its own well which serves as the family’s primary source of drinking water. All children resided in the same household since their birth. All of the parents completed a structured questionnaire asking about their child’s medical history (Supplemental Material). No parent reported that their child was ever hospitalized. Nor did any parent report that their child had a chronic illness, or experienced acute diarrheal disease (defined as 3 or more loose stools in one day). The physician who was present at recruitment did not observe any evidence of malnutrition and parents did not report that their child experienced any acute malnutrition during childhood. However, one parent reported that their child was diagnosed with pneumonia (defined as fast shallow breathing with chest indrawing) during their lifetime. None of the children were taking any medication at the time they were enrolled in this nested study. The children were breastfed for an average of 26.5 months.

Details describing the enrollment of the birth cohort have been described previously [3, 44]. Briefly, a cohort of pregnant women (N = 1,782) living in two Upazilas of Bangladesh were enrolled in a study to evaluate the effect of prenatal arsenic exposure on reproductive outcomes. Women were eligible to participate if they were ≥18 years old with a singleton pregnancy confirmed by ultrasound at the time of enrollment, would continue receiving prenatal care through Dhaka Community Hospital (DCH) affiliated clinics, used the same groundwater well as the source of their drinking water for at least the six months prior to enrollment. In this cohort, data shows that arsenic concentrations in maternal drinking water were significantly correlated with arsenic concentrations measured in cord blood (ρ = 0.47) as well as infant toenails (ρ = 0.36) and hair collected within one month of birth (ρ = 0.39) [38]. In the first recruitment phase, we included women who were <28 weeks gestation (N = 52) but in the latter two recruitment phases this criteria changed to ≤16 weeks gestation. This change in enrollment criteria arose after field teams realized they could recruit women earlier in their pregnancy which would yield a more nuanced exposure assessment during early gestation. Trained field staff orally administered questionnaires in Bangla to collect information about their medical, pregnancy, and drinking water histories. As an incentive for participation, all women were provided with free prenatal care from DCH and prenatal vitamins that were replenished during monthly checkups in the participants’ homes.

### Informed consent

All participants gave informed consent for their enrollment into the birth cohort, and for enrollment into this nested follow up study. Briefly, all study protocols underwent IRB review by Dhaka Community Hospital’s IRB and Oregon State University’s IRB board. Consent documents were translated into Bangla by a native speaker and back-translated into English by a second person fluent in Bangla to check the integrity of the translation. Consent documents were administered orally in Bangla and written informed consent was obtained by the parent or legal guardian prior to any study activity.

### Arsenic exposure

We used personal drinking water samples collected from the tubewell each mother identified as her primary source of drinking water when pregnant for our enrollment criteria.

Water was collected in a 50-ml polypropylene tubes (BD Falcon, BD Bioscience, Bedford, MA) and preserved with Reagent Grade nitric acid (Merck, Germany) to a pH <2. Total arsenic was measured using inductively coupled plasma-mass spectrometry following US EPA method 200.8 (Environmental Laboratory Services, North Syracuse, New York). The limit of detection for this method is 1 μg As/L and any sample below this level was assigned a value of 0.5 μg As/L.

### Samples for gut microbiome analysis

A fresh fecal sample was collected from the child and stored at -20°C. Samples were shipped to Oregon State University on dry ice. An aliquot of 200mg was resuspended in 1.4mL ASL buffer (Qiagen) and homogenized with 2.8mm ceramic beads followed by 0.5mm glass beads using an OMNI Bead Ruptor (OMNI International). DNA was extracted from the entire resulting suspension using QiaAmp mini stool kit (Qiagen) according to manufacturer’s protocol including optional 10 minute lysis at 90°C.

### 16S rRNA gene profiling

For the library preparation, V4 region of 16S rRNA gene was amplified using universal primers (515f and 806r) according to the published protocol [45]. Individual samples were barcoded, pooled to construct the library, and then sequenced using an Illumina Miseq (Illumina, San Diego, CA) to generate pair-ended 250 nt reads. The raw forward-end fastq files were quality-filtered, demultiplexed, and analyzed using “quantitative insights into microbial ecology” (QIIME) [46]. For quality filtering, the default parameters of QIIME were maintained in which reads with a minimal Phred quality score of <20, containing ambiguous base calls and containing fewer than 187 nt (75% of 250nt) of consecutive high-quality base calls, were discarded. Additionally, reads with three consecutive low-quality bases were truncated. The samples sequenced were demultiplexed using 12 bp barcodes, allowing 1.5 errors in the barcode. UCLUST [47] (http://www.drive5.com/uclust) was used to choose the operational taxonomic units (OTUs) with a threshold of 97% sequence similarity against the greengenes database. A representative set of sequences from each OTU were selected for taxonomic identification of each OTU by selecting the cluster seeds. The greengenes OTUs (version gg_12_10) reference sequences (97% similar) were used for taxonomic assignment using BLAST [48] with e-value 0.001. Shannon’s alpha diversity and beta diversities were calculated using QIIME. KEGG function prediction from 16S rRNA gene OTU table was performed using PICRUST galaxy server (http://huttenhower.sph.harvard.edu/galaxy/root?tool_id=PICRUSt_normalize)

### Metagenomics shotgun sequencing

The DNA extracted from stool samples was processed using Illumina Nextera XT DNA Sample Preparation Kit. 48 samples were divided into three groups (15, 16 and 17 samples per group), barcoded and sequenced in three separate lanes using Illumina HiSeq 2000 (Illumina, San Diego, CA) to produce single end 100 nucleotide reads. On average 2–18 million reads per sample were obtained. Sequences with at least one ambiguous nucleotide were filtered out by prinseq [49]. Trimmomatic [50] was used to filter out Illumina adaptor sequences (under parameters: seed mismatches:1, palindrome clip threshold: 10 and simple clip threshold: 10), to remove leading and trailing low quality bases (below quality 3), to scan the read with a 4-base wide sliding window, and to cut when the average quality per base drops below 20 and to drop reads that are below 60 bases long. Human sequences were filtered out by aligning reads against human genome (hg19) using Bowtie2 [51] under default parameters. Around 82% of original reads passed filter steps and subject to downstream analysis.

### Taxonomic/function assignment of shotgun sequencing reads

Taxonomic assignment of reads was carried out using RAPSearch2 [52] alignment against the integrated NR database. RAPSearch2 alignment hits with e-values larger than 0.001 were filtered out, and for each read, top 20 hits were retained to distinguish taxonomic groups. The taxonomical level of each read was determined by the lowest common ancestor (LCA)-based algorithm that was implemented in MEGAN4 [53]. In this algorithm, if a read had significant alignment hit in many species, it was assigned to the LCA instead of a species. Megan4 parameters were set to: min support = 1, min score = 50, min complexity = 0.44, top percent = 10, win score = 0.

SEED function assignment was performed using MEGAN4 to map reads to genes that have functional annotation in SEED database [54]. Antibiotics resistant genes sequences were retrieved from CARD database [33]. Sequence length normalized sequencing depth were generated using SOAPaligner and SOAP.coverage [55] with default parameters using downsized samples(1.3 million reads per sample).

### Metagenomics species assembly and annotation

Shotgun sequencing reads from all samples after quality control and filtration of human sequences were assembled using SOAPdenovo (Short Oligonucleotide Analysis Package)[56]. Redundant contigs were removed (shorter contigs whose 90% sequence were covered with 100% identity by a longer contig) resulting in 1,800,290 contigs. Contigs longer than 300 nucleotides were subjected to ORF prediction using MetaGene Mark [57], resulting in 1,179,372 non-redundant ORFs(95% identity covering more than 90% of a shorter redundant ORF). Abundance of ORFs are estimated by using SOAPaligner [55] to align reads to ORFs under default parameters (equal best hits are randomly assigned to one of the best hits) and using SOAP.coverage to generate ORF length normalized sequencing depth using downsized samples (1.3 million reads per sample). Co-abundant gene groups were generated using canopy clustering algorithm described previously[29]. Contigs that belong to each MGS were retrieved by their blastn similarity to an ORF in the MGS with more than 95% identity and more than 90% coverage of the ORF. Genome assembly completeness and contamination for the contigs in a MGS was assessed by CheckM [58]. Taxonomy assignment of the ORFs was performed by blastn against 5242 complete and draft bacterial genomes from NCBI FTP site under e-value 0.001. For species level assignment we required sequence identity more than 95% on more than 100bp length. A MGS was assigned to a species if more than 50% of its ORFs are assigned to a species. SEED function assignment of ORFs was generated by searching NCBI nr database using RAPSearch2 e-value 0.001 followed by MEGAN4 assignment. Arsenic metabolism gene annotation of ORFs was performed by blastx search against BacMet database predicted arsenic metabolism protein dataset (http://bacmet.biomedicine.gu.se/download/BacMet_PRE.40556.fasta). For the ORFs and contigs in Bangladeshi children cohort, we searched their homologs in the European cohort[59] by blastx and blastn against the ORF (ftp://public.genomics.org.cn/BGI/gutmeta/UniSet/UniGene.pep.gz) and contig sequences (ftp://public.genomics.org.cn/BGI/gutmeta/UniSet/UniContig.fa.gz) in the European cohort respectively.

### Quantitative PCR

Ten nanograms of DNA were used in 20 ul reactions with Fast SYBR PCR master mix (Applied Biosystems). Universal primers to amplify 16S rRNA gene were as follows: (UniF340: 5’ACTCCTACGGGAGGCAGCAGT, UniR514: 5’ATTACCGCGGCTGCTGGC). Primers for arsenic resistance genes were: ArsC F: 5’TGCCGATATGGGGATTTCCG, ArsC R: 5’AGCGTTTACCCGCTTCATCA (product length 275 bp); ArsB F: 5’CGCAGATTTCTTTGGCCTCG, ArsB R: 5’AATCGCAGCCAATCACGTTG (product length 620 bp). StepOne Plus real-time PCR instrument was used with standard fast cycle conditions (Applied Biosystems). Data were normalized to amounts of total bacterial DNA estimated by universal primers and expressed as 2^deltaCt between each sample and the median Ct of all samples.

### Statistical analyses

Descriptive statistics including two-tailed t-tests, Wilcoxon signed-rank test, and chi-square tests were used to examine the differences of selected characteristics between the high and low abundance group. Differential abundance of bacterial taxonomic groups between high exposure and low exposure groups were analyzed for each taxonomic level using Mann–Whitney U test implemented in QIIME (group_significance.py) considering taxa with >1% of abundance [60].

For analysis of metagenomics shotgun data (i.e. SEED annotated bacterial genes), we used similar method by detecting differentially abundant taxa followed by correlation analysis. We then integrated results of class comparison and correlation by calculating Fisher's combined probability test [61]. We then selected those genes presenting concordance between direction of fold-change and sign of correlation (e.g. enriched in high exposure group and positively correlated to arsenic levels) and tested them for significance using Fisher's combined probability test. The multiple hypotheses testing correction was performed by estimating false discovery rate via Benjamini-Hochberg method [62].

For SEED level 2 functional category enrichment analysis (Fig 3), we compared frequencies of SEED level 2 functional categories in the 332 SEED genes to those in all SEED genes detected in the samples. For example, we calculated the number of SEED genes under Function A in 332 gene set and the number of genes under function A in all SEED genes. The corresponding counts for all non-function A SEED level 2 categories were also generated to create a 2x2 contingency table and test relationship between function A and the 332 SEED gene set using chi-square test.

Chi-squared test was also used to measure the significance of overlap between arsenic-related genes and genes enriched by antibiotics treatment. Specifically, for all the SEED genes detected in arsenic study samples (5870 SEED genes), we calculated a 2x2 contingency table recording the numbers of genes in/out of the 332 gene set that correlates with arsenic and the numbers of genes enriched/not enriched in 1689 SEED genes enriched in antibiotic-treated mice [32]. For testing if a metagenome species (MGS) is a confounding factor, we used Cochran-Mantel-Haenszel Chi-Squared Test implemented in R function mantelhaen.test.

## Supporting information

S1 Fig Analysis of alpha and beta diversity of microbiota. (TIFF)

S2 Fig Correlation between taxa and arsenic exposure. (TIFF)

S3 Fig Workflow of reference free assembly of genomes. (TIFF)

S4 Fig Simulation of different genome assembly completeness for MGS0010. (TIFF)

S5 Fig Simulation of different genome assembly completeness for MGS0010 by p-value. (TIFF)

S6 Fig Antibiotic resistance genes by arsenic exposure. (TIFF)

S7 Fig Abundance of arsenic resistance genes in high arsenic exposure group. (TIFF)

S8 Fig Taxa abundance estimates between 16S RNA-based and whole genome shotgun sequencing. (TIFF)

S9 Fig Arsenic resistance operons in assembled contigs. (TIFF)

S10 Fig UniBac qPCR for total bacterial load. (TIFF)

S1 Table Abundance of KEGG functions between high and low arsenic exposure groups. (XLSX)

S2 Table Abundance of bacterial genes between high and low arsenic exposure groups with differential abundance (two-tailed P <0.1). (XLSX)

S3 Table Assembled metagenomics species (MGS) from sampled population. (XLSX)

S4 Table Arsenic-enriched genes found in the bacteria that survived antibiotic treatment in mouse gut. (XLSX)

S5 Table Contigs that harbor arsenic resistance operons identified through blastn search against NCBI nucleotide database. (XLSX)

S6 Table Antibiotic resistance genes identified using Comprehensive Antibiotic Resistance Database (CARD) database. (XLSX)

S7 Table Assemblage of 100 large genomes in the genome with open reading frames (ORF) annotated with SEED functions. (XLSX)

S8 Table Genome MGS0010 which showed a significant tendency for enrichment within the 332 arsenic related SEED functions. (XLSX)

S1 Questionnaire Basic questionnaire. (DOC)

## Abbreviations

MetaHIT: Metagenomics of the Human Intestinal Tract
FDR: False discovery rate
KEGG: Kyoto Encyclopedia of Genes and Genomes
MGS: metagenomic species
ORFs: Open Reading Frames
OTU: operational taxonomic units
PICRUSt: Phylogenetic Investigation of Communities by Reconstruction of Unobserved States
MEGAN 4: MEtaGenome Analyzer
CARD: The Comprehensive Antibiotic Resistance Database
QIIME: Quantitative insights into microbial ecology
SOAP: Short Oligonucleotide Analysis Package
