# Supplementary figures and images for: Arsenic exposure and intestinal microbiota in children from Sirajdikhan, Bangladesh
Source: PLoS One. 2017 Dec 6;12(12):e0188487. doi: 10.1371/journal.pone.0188487 (PMC5718612; doi:10.1371/journal.pone.0188487)

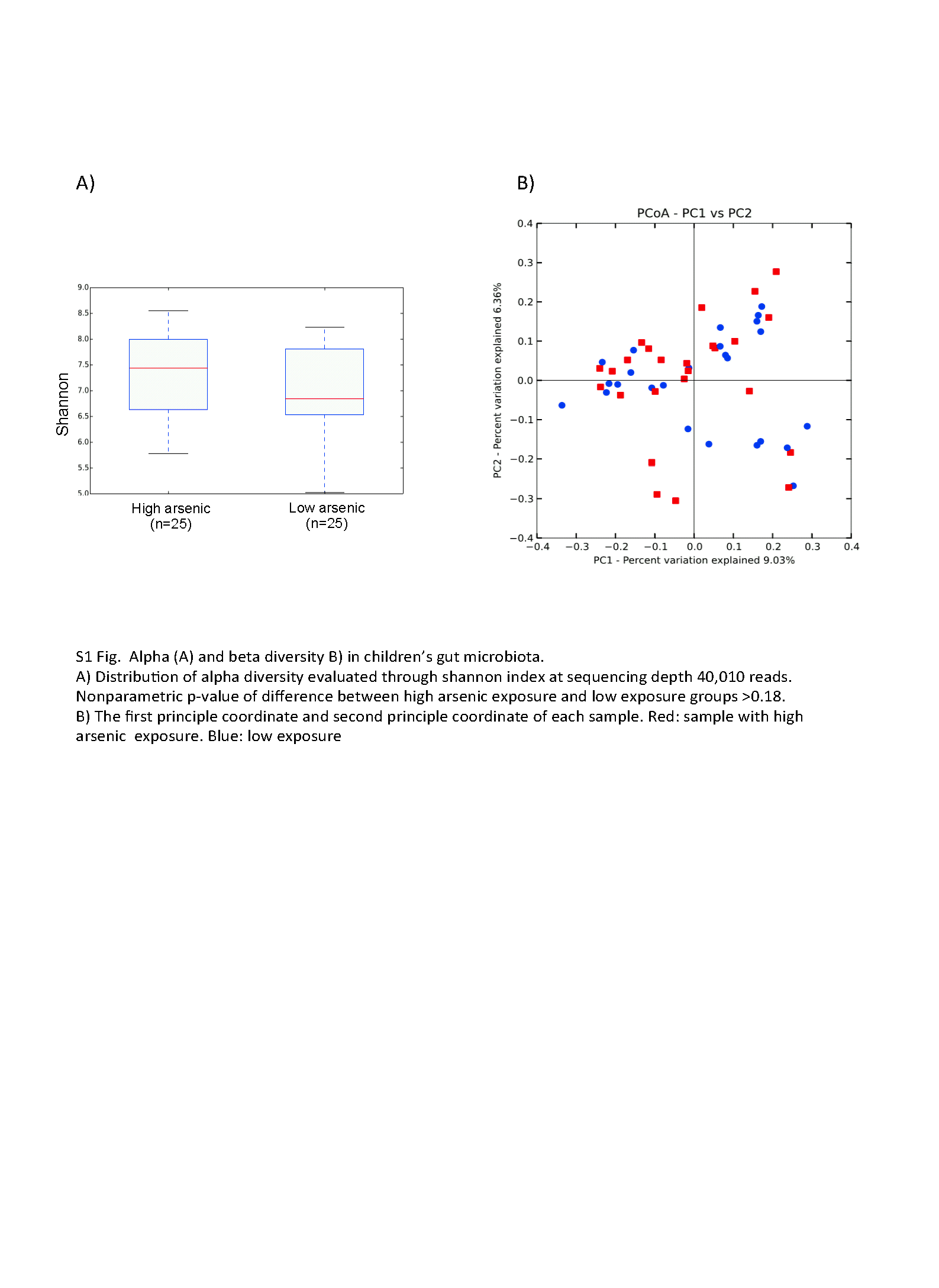

Supplement: S1 Fig — (TIFF) [file pone.0188487.s001.tiff]

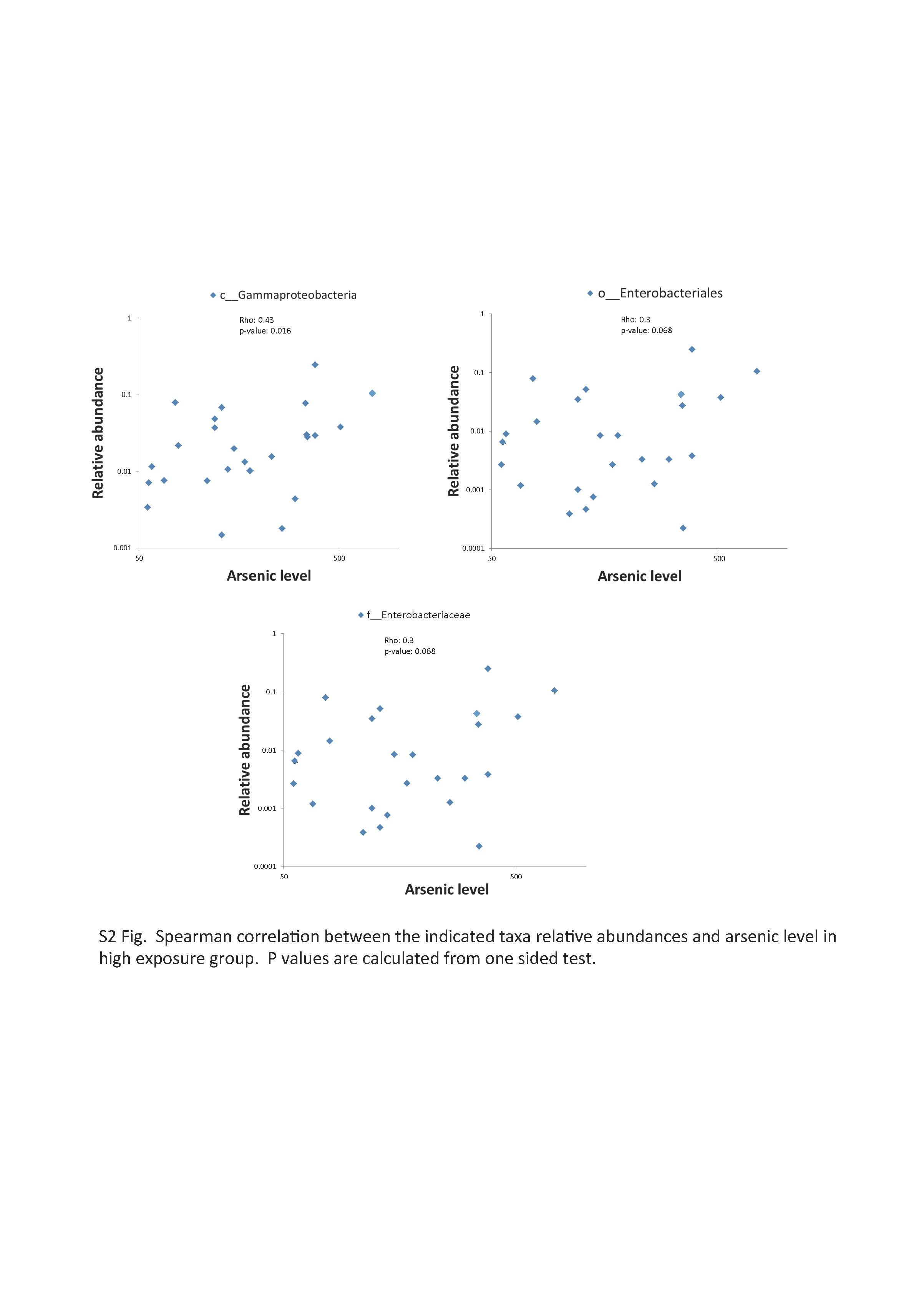

Supplement: S2 Fig — (TIFF) [file pone.0188487.s002.tiff]

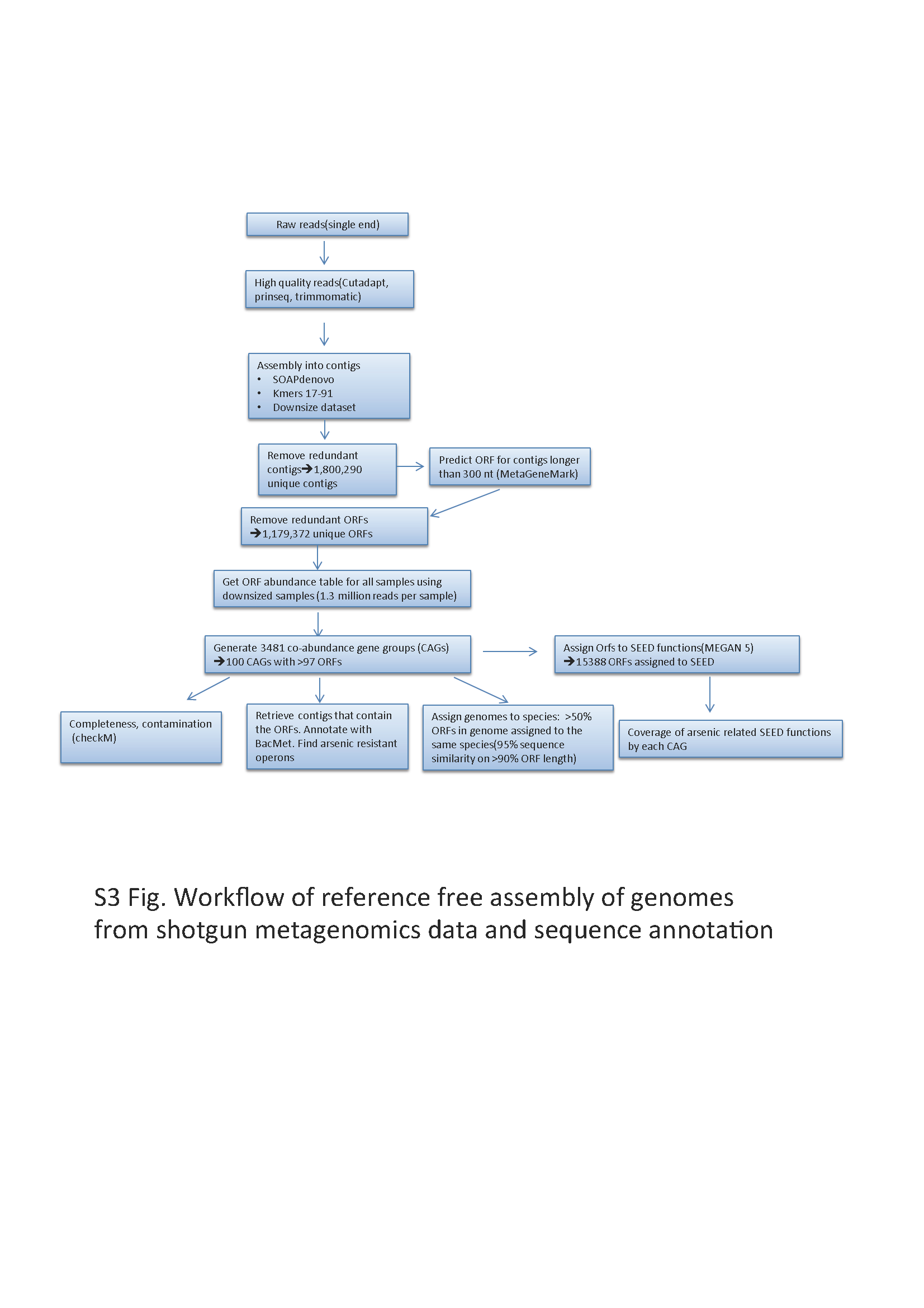

Supplement: S3 Fig — (TIFF) [file pone.0188487.s003.tiff]

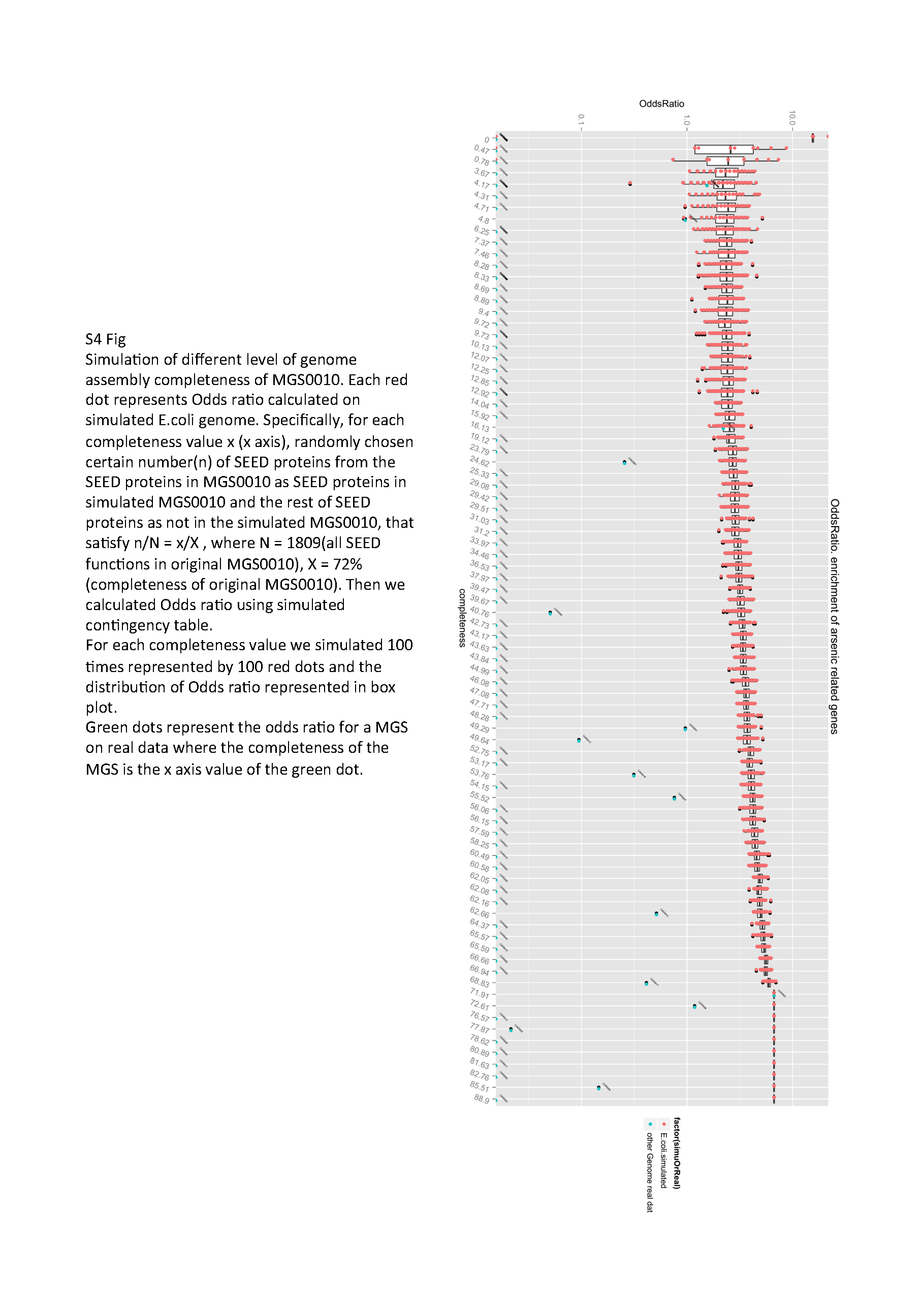

Supplement: S4 Fig — (TIFF) [file pone.0188487.s004.tiff]

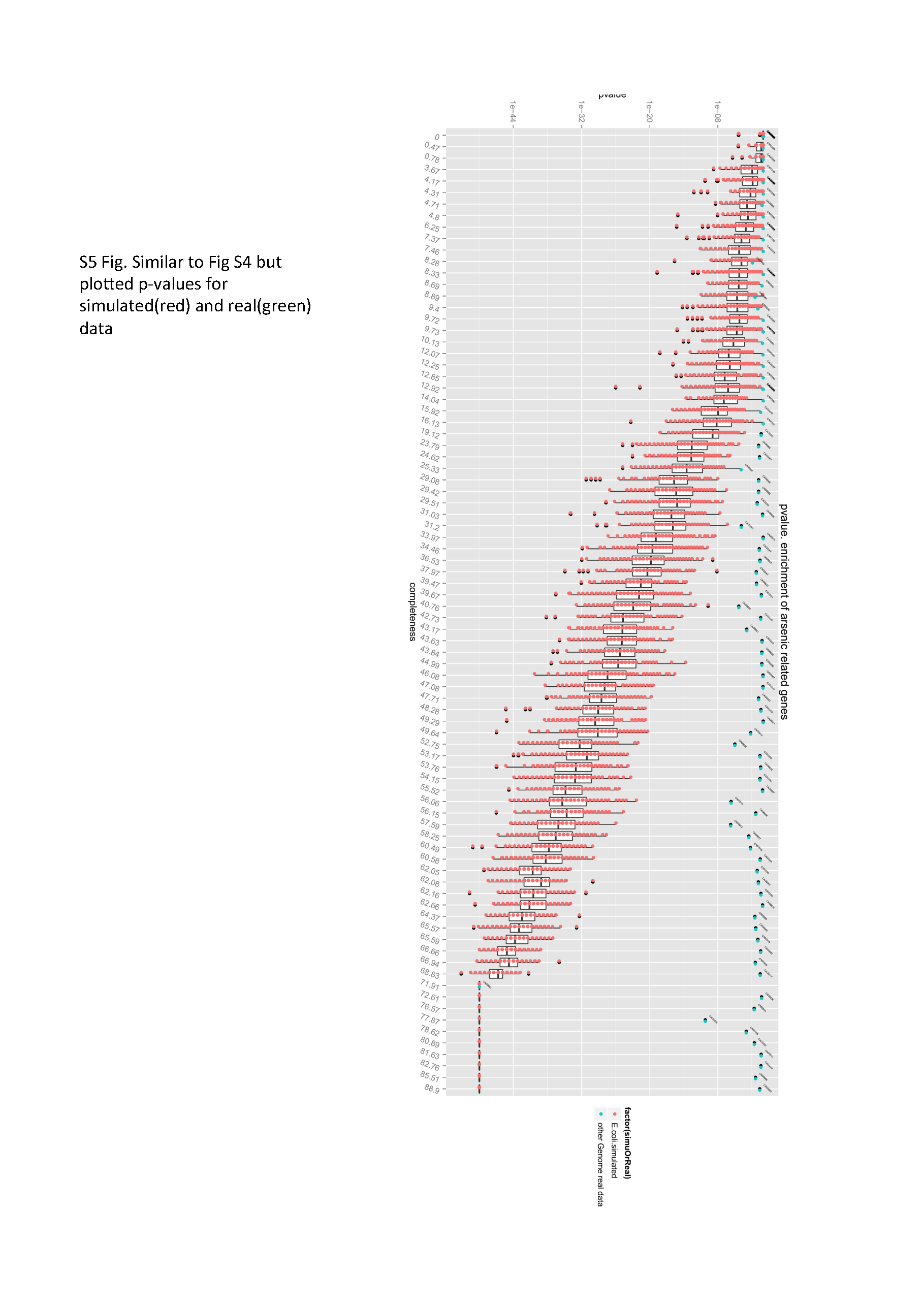

Supplement: S5 Fig — (TIFF) [file pone.0188487.s005.tiff]

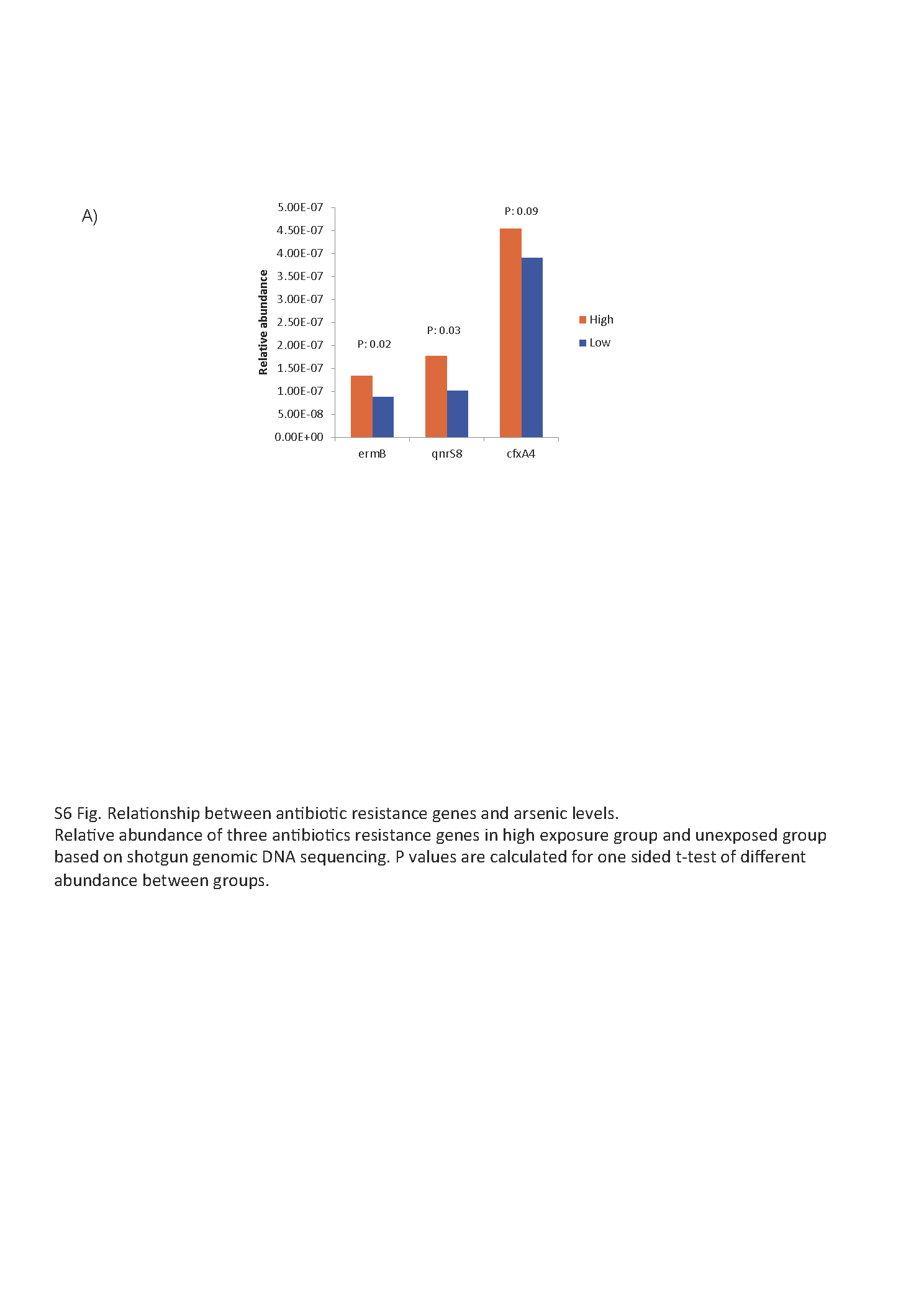

Supplement: S6 Fig — (TIFF) [file pone.0188487.s006.tiff]

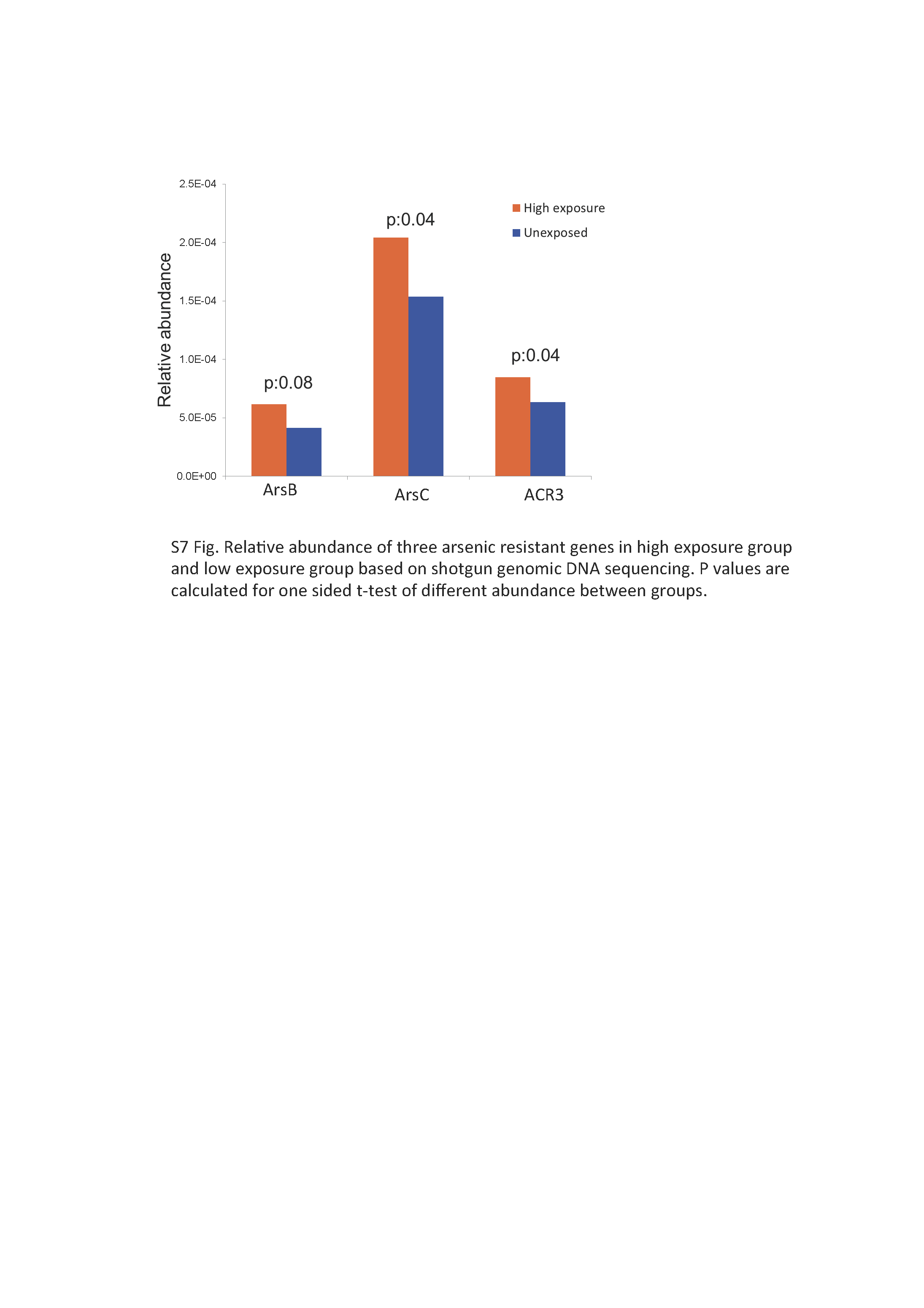

Supplement: S7 Fig — (TIFF) [file pone.0188487.s007.tiff]

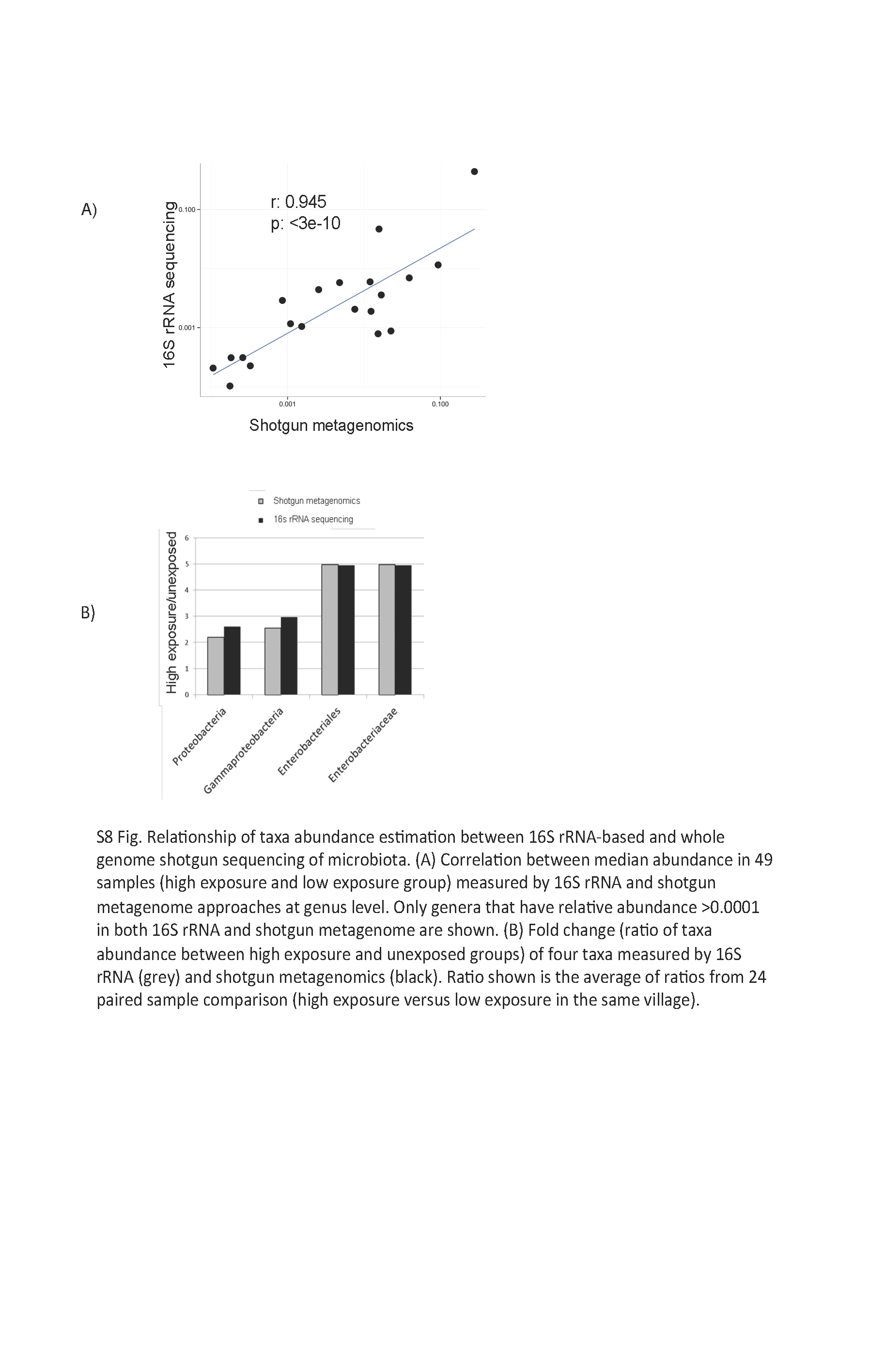

Supplement: S8 Fig — (TIFF) [file pone.0188487.s008.tiff]

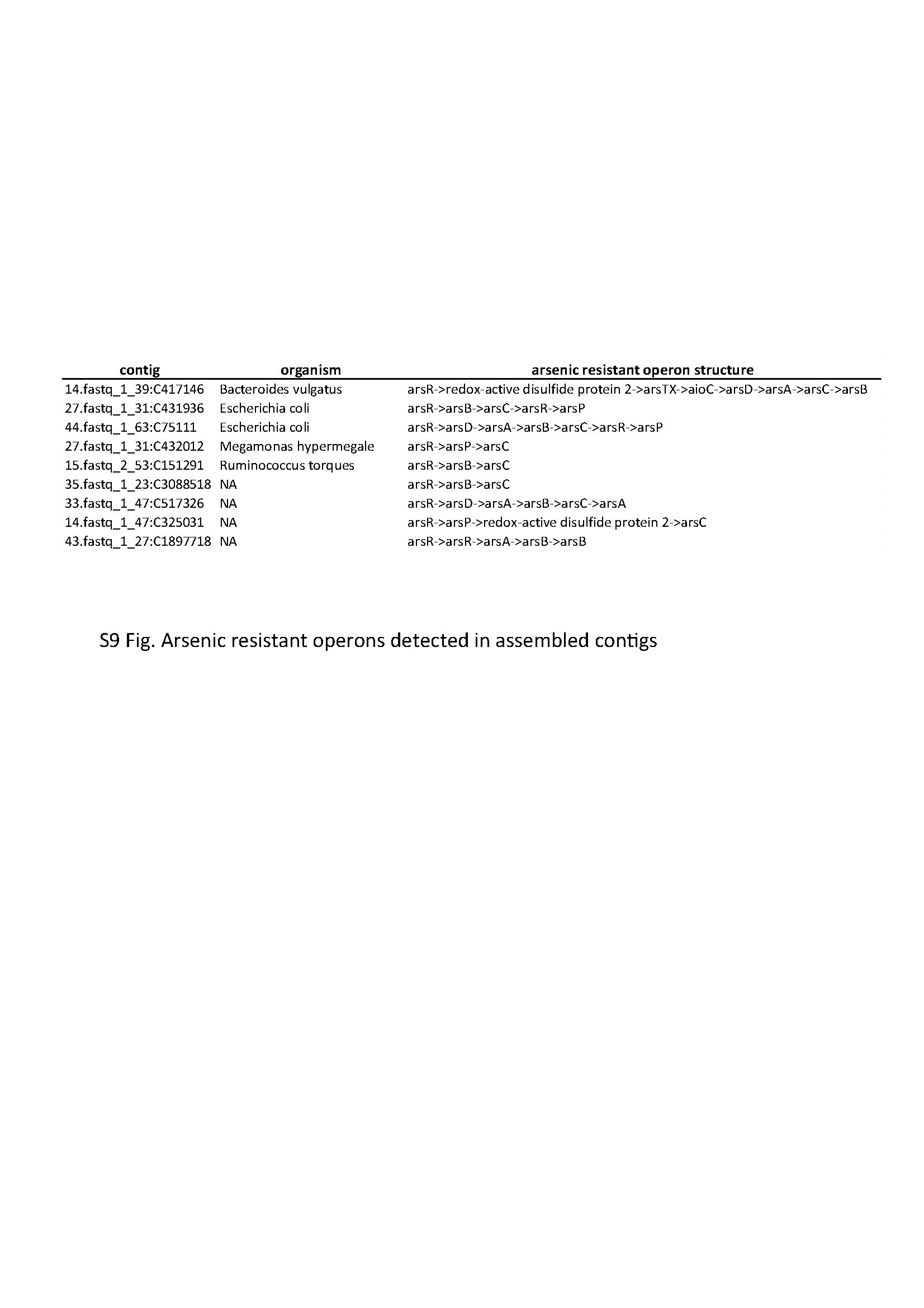

Supplement: S9 Fig — (TIFF) [file pone.0188487.s009.tiff]

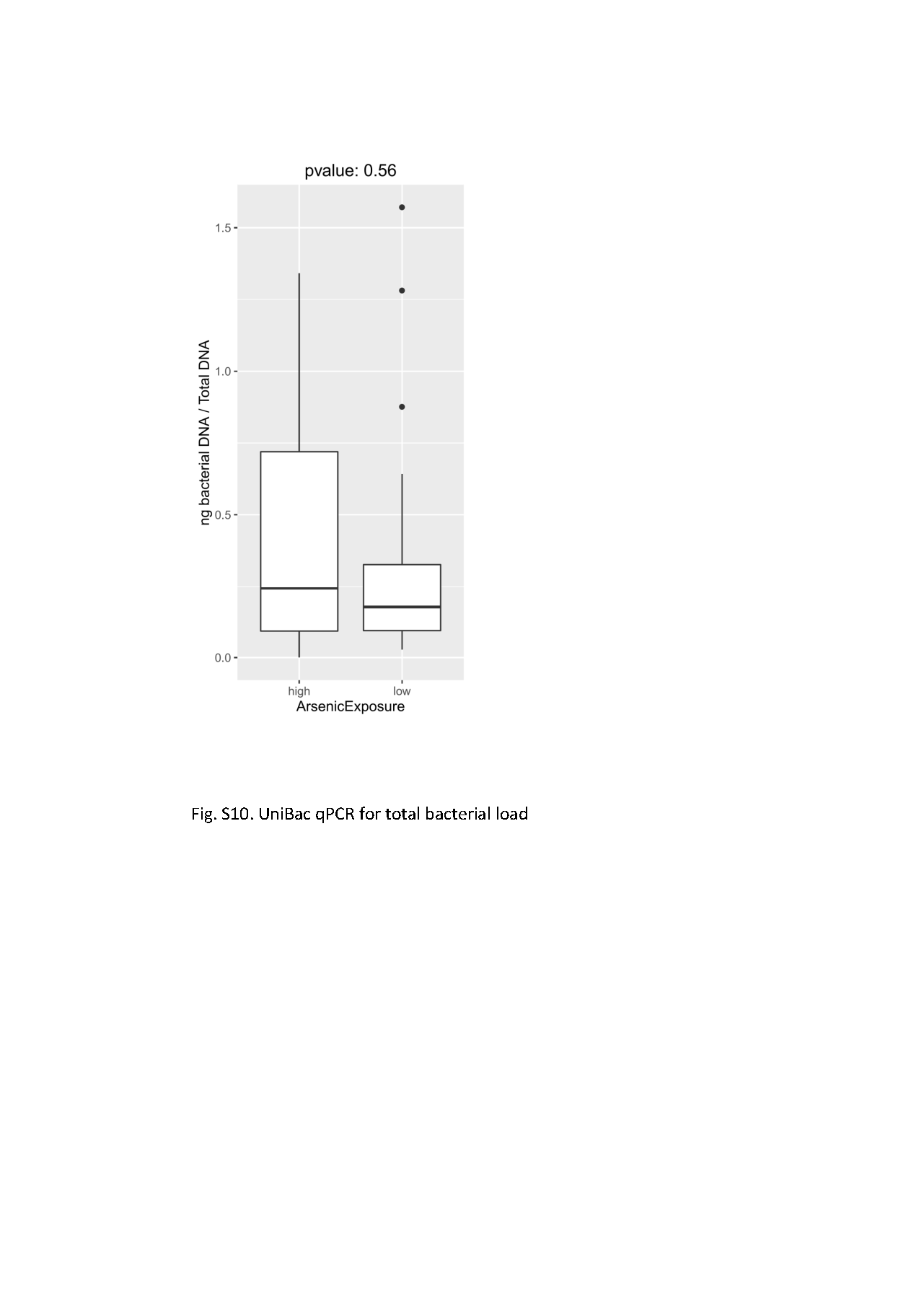

Supplement: S10 Fig — (TIFF) [file pone.0188487.s010.tiff]
