# Supplementary material for: Arsenic exposure and intestinal microbiota in children from Sirajdikhan, Bangladesh
Source: PLoS One. 2017 Dec 6;12(12):e0188487. doi: 10.1371/journal.pone.0188487 (PMC5718612; doi:10.1371/journal.pone.0188487)
Supplement: S1 Questionnaire — (DOC) [file pone.0188487.s019.doc]

**S1 Questionnaire. Basic Questionnaire**

**INSTRUCTIONS FOR INTERVIEWER:**

- Please read ALL response categories for a question before accepting an answer from the participant.
- Circle the most appropriate response from the participant or fill in the blank space when an open-ended question is asked.
- If the participant says she does not know the answer, please circle DK.
- If she refuses to answer, circle R.
- If the participant is hesitant about answering a particular question, prompt her until she selects a response.

**PHYSICAL EXAMINATION**

Q1. Height: __ __ __.__ __ cm

Q2. Weight: __ __.__ __ kg

Q3. Mid-arm Circumference __ __**.**__ __ cm

Q4. Blood pressure: ______/______ mmHg

Q5. Evidence of Marasmus (non-oedematous malnutrition): yes no (circle one)

Q6. Evidence of Kwashiorkor or Pustihin (oedematous malnutrition): yes no (circle one)

Q7. Evidence of Oedema in both feet: yes no (circle one)

Q8. Presence of skin lesions or other evidence of arsenic poisoning (circle as many as apply):

None Leucomelanosis Melanosis Keratosis Hyperatosis Other: ___________

**MEDICAL HISTORY:**

Q9. Baby's date of birth (dd/mm/yyyy) __ __ __/__ __/2 0 __ __

Q10. Today’s date (dd/mm/yyyy) __ __ __/__ __/2 0 __ __

Q11 Child’s sex: Male Female (circle one)

Q12. Birth order of subject (child) 1^st^/2^nd^/3^rd^/4^th^/5^th^/6^th^ /7^th^ /8^th^/9^th^/10^th^/other (circle one)

Q13. Has your child ever been admitted to the hospital? Yes/ No/ DK / R (circle one)

*If yes, go to 5a and ask questions for each hospital admission starting with the most recent hospital admission*

| *1^st^ admission* | *2^nd^ admission* | *3^rd^ admission* | *4^th^ admission* |
| --- | --- | --- | --- |
| Q13a. How old was your child when they were admitted to the hospital?  ______ (months) | Q13d. How old was your child when they were admitted to the hospital?  ______ (months) | 1Q3g. How old was your child when they were admitted to the hospital?  ______ (months) | Q13j. How old was your child when they were admitted to the hospital?  ______ (months) |
| Q13b. Why was your child admitted to the hospital?   1. Fever 2. Diarrhea 3. Injury 4. Other | Q13e. Why was your child admitted to the hospital?   1. Fever 2. Diarrhea 3. Injury 4. Other | Q13h. Why was your child admitted to the hospital?   1. Fever 2. Diarrhea 3. Injury 4. Other | Q13k. Why was your child admitted to the hospital?   1. Fever 2. Diarrhea 3. Injury 4. Other |
| Q13c. What was the specific cause of the hospital admission?  ________________  ________________  *Note:* *Write DK if the specific cause is not known* | Q13f. What was the specific cause of the hospital admission?  _______________  ________________ *Note:* *Write DK if the specific cause is not known* | Q13f. What was the specific cause of the hospital admission?  _______________  ________________ *Note:* *Write DK if the specific cause is not known* | Q13f. What was the specific cause of the hospital admission?  _______________  ________________ *Note:* *Write DK if the specific cause is not known* |

Q14. Has your child ever been diagnosed by a doctor with any chronic illness or disease? Yes/ No/ DK / R (circle one)

14a. If yes, what are the diagnosed illnesses or diseases?

______________________________________________________________

______________________________________________________________

______________________________________________________________

______________________________________________________________

______________________________________________________________

Q15. Is your child currently taking any medications? Yes/ No/ DK / R (circle one)

Q15a. If yes, what kind of medications are they currently taking?

(check the box for all that apply)

- - Anti-convulsant
  - Anti-inflammatory/Non-steroidal
  - Brochodilator
  - Antibiotic
  - Traditional or Ayurvedic medicine
  - Other

Q16. Has your child received any immunizations? Yes/ No/ DK / R (circle one)

16a. If yes, ask for the vaccination booklet and copy record into table

|  | 1st | 2nd | 3rd | 4th | 5th |
| --- | --- | --- | --- | --- | --- |
| BCG | Due date:  Given date: | Due date:  Given date: | Due date:  Given date: | Due date:  Given date: | Due date:  Given date: |
| Penta (DPT, hepB, HIB) | Due date:  Given date: | Due date:  Given date: | Due date:  Given date: | Due date:  Given date: | Due date:  Given date: |
| Polio | Due date:  Given date: | Due date:  Given date: | Due date:  Given date: | Due date:  Given date: | Due date:  Given date: |
| MR | Due date:  Given date: | Due date:  Given date: | Due date:  Given date: | Due date:  Given date: | Due date:  Given date: |
| Measles | Due date:  Given date: | Due date:  Given date: | Due date:  Given date: | Due date:  Given date: | Due date:  Given date: |

Q17. Has your child experienced acute diarrhea? Yes/ No/ DK / R (circle one)

[*Definition of acute diarrhea: passage of 3 or more loose or liquid stools per day*]

17A. If yes, did your child every have blood in their diarrhea? Yes/No/DK/R (circle one)

17B. How many times has your child seen a health care worker or visited a hospital because of diarrhea? _____________ (number of visits in past week)

_____________ (number of visits in past month)

_____________ (number of visits in past year)

Q18. Has your child experienced a respiratory infection? Yes/ No/ DK / R (circle one)

[*Definition of respiratory infection is rapid respiration and chest in-drawing, cough and/or difficulty breathing combined with rapid respiration*]

Q18A. How many times has your child seen a health care worker or visited a hospital because of respiratory infection? _____________ (number of visits in past week)

_____________ (number of visits in past month)

_____________ (number of visits in past year)

Q19. Has your child ever been diagnosed with pneumonia? Yes/ No/ DK / R (circle one)

Q20. Does your child put non-food items (e.g., dirt, grass) in his/her mouth more often than other children? Yes/ No/ DK/ R (circle one)

**FEEDING and DRINKING WATER**

Q21. Did you breastfeed your child? Yes /No /DK /R (circle one)

Q22. How long did you breastfeed your child? ___________ (months)

Q23. How old was your child when you introduced any foods or fluids (besides breast milk) to their diet? ___________ (months)

Q24. Does your child drink water? Yes /No /DK /R (circle one)

24a. If yes, how many cups of water per day (250mls)? ________ DK /R

Q26. What type of water source do you use for drinking now?

1. River/Pond without filtration
2. River/Pond that is filtered
3. Rain water
4. Shallow dug well
5. Tube well (Deep)
6. Tube well (Shallow)
7. Don’t Know
8. Refused

Q27. Have you changed your source of drinking water since you’ve given birth?

Yes /No /DK /R (circle one)

**HOUSEHOLD CHARACTERISTICS**

Q28. Is your child exposed to smoke from cooking fires? Yes /No /DK /R (circle one)

*[Exposed is defined as more than just walking past cooking fires.]*

Q29. Does your child help cook? Yes /No /DK /R (circle one)

Q30. Do you have kerosene lamps or heaters in your house? Yes /No /DK /R (circle one)

Q31. Does anyone smoke tobacco inside your house? Yes /No /DK /R (circle one)

**24 HOUR DIETARY RECALL:**

- Ask mother or caregiver “Please tell me what your child has eaten **in the last 24 hours**. That is, from yesterday morning when he or she woke up until this morning.”
- Record the time, name of food an amount in the table below.
- Display the utensils when administering the questionnaire to ensure accuracy
- For the amount of food, use the following codes for normally used serving items:

| ST= small teaspoon  LT= large teaspoon  L = dal/soup ladle  SS = small serving spoon | LS = large serving spoon  RS = rice spoon  SB = small bowl  LB = large bowl  P = plate |
| --- | --- |

For protein items, use your thumb as a serving amount (1 thumb ~ 1 ounce)

For fruit or other item, just list number of pieces consumed.

| Time | Food list | Amount of food |
| --- | --- | --- |
| *Example:* |  |  |
| *06:00* | *Rice* | *½ RS* |
|  |  |  |
|  |  |  |
|  |  |  |
|  |  |  |
|  |  |  |
|  |  |  |
|  |  |  |
|  |  |  |
|  |  |  |
|  |  |  |
|  |  |  |
|  |  |  |
|  |  |  |
|  |  |  |
|  |  |  |
|  |  |  |
|  |  |  |
|  |  |  |
|  |  |  |
